# Healthcare utilization and adverse outcomes stratified by sex, age and long-term care residency using the Alberta COVID-19 Analytics and Research Database (ACARD): a population-based descriptive study

**DOI:** 10.1186/s12879-023-08326-5

**Published:** 2023-05-19

**Authors:** Elissa Rennert-May, Alysha Crocker, Adam G. D’Souza, Zuying Zhang, Derek Chew, Reed Beall, David M. Vickers, Jenine Leal

**Affiliations:** 1 Department of Medicine, University of Calgary, Calgary, AB Canada; 2 Department of Community Health Sciences, University of Calgary, Calgary, AB Canada; 3 O’Brien Institute for Public Health, University of Calgary, Calgary, AB Canada; 4 Department of Microbiology, Immunology and Infectious Diseases, University of Calgary, Calgary, AB Canada; 5 Snyder Institute for Chronic Diseases, University of Calgary, Calgary, AB Canada; 6 Alberta Health Services, Calgary, AB Canada; 7 Centre for Health Informatics, University of Calgary, Calgary, AB Canada; 8 Department of Cardiac Sciences, University of Calgary, Calgary, AB Canada; 9 Mozell Core Analysis Lab, Centre for Health Informatics, University of Calgary, Calgary, AB Canada

**Keywords:** COVID-19, Population-based epidemiology, Long-term care

## Abstract

**Background:**

Understanding the epidemiology of Coronavirus Disease of 2019 (COVID-19) in a local context is valuable for both future pandemic preparedness and potential increases in COVID-19 case volume, particularly due to variant strains.

**Methods:**

Our work allowed us to complete a population-based study on patients who tested positive for COVID-19 in Alberta from March 1, 2020 to December 15, 2021. We completed a multi-centre, retrospective population-based descriptive study using secondary data sources in Alberta, Canada. We identified all adult patients (≥ 18 years of age) tested and subsequently positive for COVID-19 (including only the first incident case of COVID-19) on a laboratory test. We determined positive COVID-19 tests, gender, age, comorbidities, residency in a long-term care (LTC) facility, time to hospitalization, length of stay (LOS) in hospital, and mortality. Patients were followed for 60 days from a COVID-19 positive test.

**Results:**

Between March 1, 2020 and December 15, 2021, 255,037 adults were identified with COVID-19 in Alberta. Most confirmed cases occurred among those less than 60 years of age (84.3%); however, most deaths (89.3%) occurred among those older than 60 years. Overall hospitalization rate among those who tested positive was 5.9%. Being a resident of LTC was associated with substantial mortality of 24.6% within 60 days of a positive COVID-19 test. The most common comorbidity among those with COVID-19 was depression. Across all patients 17.3% of males and 18.6% of females had an unplanned ambulatory visit subsequent to their positive COVID-19 test.

**Conclusions:**

COVID-19 is associated with extensive healthcare utilization. Residents of LTC were substantially impacted during the COVID-19 pandemic with high associated mortality. Further work should be done to better understand the economic burden associated with related healthcare utilization following a COVID-19 infection to inform healthcare system resource allocation, planning, and forecasting.

## Background

The Coronavirus Disease of 2019 (COVID-19) has had a substantial impact globally and has negatively impacted human health and the global economy [1]. This disease has also been associated with significant loss of life [1]. An understanding of the epidemiology, and healthcare resource utilization of COVID-19 in a local context is critically important for future pandemic preparedness and planning for possible increases in case volume, particularly associated with escalations in variant strains. Additionally, as with many other infections, once epidemiological risk factors for adverse outcomes including hospitalization and invasive mechanical ventilation (IMV) are identified, preventative and targeted interventions are better able to be implemented [2]. This will potentially aid in mitigating some of the negative outcomes associated not only with COVID-19 but also with other emerging respiratory viruses.

There have been several studies in Canada exploring the epidemiology of COVID-19 and health resource utilization [3, 4]. Work exploring COVID-19 in Ontario from March to September 2020 demonstrated a shift from older populations most frequently being diagnosed (> 60 years) to younger populations (10–39 years). It was also found that about 10% of patients with COVID-19 required hospitalization and 22% of those patients ended up in intensive care units (ICU) with length of stay (LOS) averaging 20.5 days for those that required IMV [3]. More recently, a paper explored the risk of death and unplanned readmission after discharge from a COVID-19 hospitalization in Alberta and Ontario within 30 days [5]. Between January 1, 2020 and September 30, 2021 the authors found that death and re-admission were common and that risk assessment should include consideration for multiple variables including sex and Charlson Comorbidity Index [5]. Comorbidity indexes such as the aforementioned Charlson score or the Elixhauser index have been utilized to establish prognostic scoring models which can assist with management of patients according to their prognosis scores [6].

There has also been work done exploring COVID-19 and its outcomes in patients living in long-term care (LTC) facilities [7]. While the impact of COVID-19 on LTC residents varied through the timeline of this pandemic, partially due to the protection offered by vaccination, this population suffered disproportionately from COVID-19 illness with excess mortality [7, 8]. Recent work on LTC populations in Ontario found that of residents in LTC the 30-day mortality following a positive COVID-19 test was substantially elevated at 28.7% [9].

Much of the previous work on COVID-19 has focused on patients who required hospitalization and subsequent outcomes including readmissions [10, 11]. Previous epidemiological summaries suggest differences in geography and patient health demographics influence the impact of COVID-19 [12], highlighting the importance of local population-based research. Therefore, our study was designed to address this gap in the literature and more broadly assess outcomes associated with COVID-19 cases in Alberta from a population-based perspective, regardless of hospitalization. In the current study we investigated all adult patients (≥ 18 years of age) who tested positive for COVID-19 from March 1, 2020 to December 15, 2021. We primarily sought to describe age- and sex-stratified outcomes of hospitalization rate, ambulatory visits to the emergency department or urgent care (i.e. unplanned) and all-cause mortality. For secondary outcomes we assessed healthcare utilization amongst those hospitalized including ICU admissions during hospitalization, length of stay (LOS), IMV, and in-hospital mortality.

## Methods

### Study Design

A multi-centre, retrospective population-based descriptive study using secondary data sources in Alberta, Canada.

### Patient Population

All adult patients (≥ 18 years of age) tested and subsequently positive for COVID-19 from March 1, 2020 to December 15, 2021. Only the first incident case of COVID-19 per patient was included. A positive case was defined as those with a laboratory confirmation of infection with the virus that causes COVID-19. This consisted of either a positive nucleic acid amplification test (NAAT) on at least two specific genomic targets or a single positive target with the nucleic acid sequencing at a Provincial Public Health Laboratory where NAAT tests have been validated (or a confirmed positive result by the National Microbiology Laboratory by NAAT).

### Data sources

The Alberta COVID-19 Analytics and Research Database (ACARD) is a population-based database using secondary data sources to understand local epidemiology and resource use implication of COVID-19. It includes public health, clinical, laboratory, pharmacy, administrative and financial data. It was developed in collaboration with Alberta Health Services’ Data and Analytics department and the University of Calgary’s Centre for Health Informatics. Data linkages between different sources used deterministic matching on the patients’ personal healthcare number (PHN), date of birth, and sex. A waiver of consent was obtained as part of the ethics application for this work in order to collect PHNs required for data linkage, as patients could not be anonymized to complete this. onymization was done following data linkage prior to releasing the data to researchers. The following data sources were used for this study:

#### Alberta Precision Laboratories (APL)

All laboratory test results collected in Alberta that were positive for COVID-19 were provided by APL.

#### AHS Enterprise Data Warehouse (EDW)

The AHS EDW was utilized to access information from the Discharge Abstract Database (DAD), the National Ambulatory Care Reporting System (NACRS), Vital Statistics, the Alberta Continuing Care Information System (ACCIS), Practitioner Claims, and the Paris and Meditech medical information systems. Incident cases of COVID-19 were linked to these data assets to categorize those cases that were collected in hospital (DAD), the Emergency Department (NACRS) and long-term care (ACCIS) Tests not collected in those settings were assumed to be community patients.

### Definitions

Patient demographics (i.e., sex and age) were provided from all sources to confirm accuracy. Long-term care residents were defined as those individuals registered in ACCIS at either a long-term care facility or designated supportive living level 3, 4 or 4-Dementia facility at the time of their COVID-19 collection date. Elixhauser comorbidities were defined using the *International Statistical Classification of Diseases and Related Health Problems, Tenth Revision, Canada* (ICD-10-CA) diagnosis codes from DAD admissions and NACRS visits, and ICD-9 diagnosis codes from Practitioner Claims visits in the 2 years prior to the COVID-19 collection date [13].

For the primary outcomes of hospitalizations, ambulatory visits and all-cause mortality, DAD was used to identify previous hospitalizations, defined as having a discharge date within 1 year prior to the COVID-19 collection date. A 60-day hospitalization outcome was defined as non-elective acute care admissions occurring either 24 h before or within 60 days of the COVID-19 collection date to identify patients hospitalized following their COVID-19 diagnosis. The all-cause mortality measure was defined as death due to any cause within 60 days of the collection date of COVID-19 and retrieved from Alberta Vital Statistics. Patient status at 60 days was used to describe the status of each COVID-19 case on the 60th day from the COVID-19 collection date. Patients were categorized as either deceased, hospitalized (non-ICU), hospitalized (ICU/cardiovascular (CV)-ICU), in LTC, or discharged from the hospital. NACRS was utilized to identify ambulatory visits. A 60 day follow up was chosen to be consistent with prior literature and to allow for comparisons with existing published data [14].

For the secondary outcomes of in-hospital resource utilization including ICU admissions, IMV, and LOS, the following methods were utilized. Up to six special care unit admissions (i.e., ICU, Cardiac critical care units) in hospitalizations within 60 days of the COVID-19 collection date were provided from DAD. The IMV outcome was defined as those patients with an interventional code in DAD for invasive ventilation (i.e., 1.GZ.31) within 60 days of the collection date of the positive COVID-19 test. The hospital length of stay (LOS) outcome was defined as the difference in days between the discharge and admission date in DAD for the first acute care hospitalization within 60 days of the COVID-19 collection date. The in-hospital mortality outcome within 60 days of the COVID-19 collection date was defined by the disposition code for death in hospitalizations (72 and 74), found within DAD.

### Statistical analyses

Descriptive statistics including frequencies and percentages for categorical variables and means with standard deviations (SD) for normally distributed continuous variables and medians with interquartile ranges (IQR) for skewed continuous variables were used to describe characteristics of COVID-19 cases. Data were stratified by sex and LTC residency. Kaplan Meier curves were used to visually represent the cumulative proportion of cases’ time to acute care hospitalization or death, stratified by LTC residency. Chi-squared analyses were used to compare mortality rates in LTC versus non LTC residents stratified by age.

We used adjusted modified Poisson models to explore variables of age, sex, number of hospitalizations in year prior to diagnosis of COVID-19, and comorbidities associated with poor outcomes in LTC residents. Poor outcomes included mortality, hospitalization, ICU admission and mechanical ventilation within 60 days.

All statistical analyses were done using STATA/SE version 16 software (StataCorp, College Station, TX). Ethics for this study was granted by the University of Calgary health research ethics board (REB20-0688). The data used for this study is not publicly available as it released by AHS. However, this data can be obtained through appropriate ethics application and liaison with AHS.

## Results

Between March 1, 2020 and December 15, 2021, there were 255,037 adult individuals with laboratory-confirmed COVID-19 in Alberta, resulting in a provincial incidence proportion of 5,739 cases per 100,000 population. The median age was 39 years (IQR 23) with 50.4% of cases among females. Of the confirmed cases, 84.2% were among those under the age of 60 years.

Of the total confirmed COVID-19 cases, 4,809 (1.9%) were residents of LTC with a median age of 85 years (IQR 15). In LTC, 61.6% of cases occurred among females. 6% of confirmed COVID-19 cases among non-LTC residents had at least one previous hospitalization in the previous year, compared to 31.9% of cases among LTC residents. Male residents had more previous hospitalizations than female residents in LTC (36.5% vs. 29.0%). Depression, as a comorbidity, was most common in individuals with COVID-19 and observed most among females (22.6% vs. 16.6% in males) (Table 1). Among LTC residents, the proportion of comorbidities between males and females were similar.

**Table 1:** Demographic characteristics of all incident cases of COVID-19 between March 1, 2020 and December 15, 2021, stratified by sex and LTC residency

	All Individuals (N = 225,037)			Individuals in LTC (N = 4,809)		Individuals not in LTC (N = 250,228)		
Age Group (years)								
	Male (N = 126 337)	Female (N = 128 607)	Not Documented (N = 93)	Male (N = 1845)	Female (N = 2964)	Male (N = 124 492)	Female (N = 125 643)	Not Documented (N = 93)
18–29	33 211 (26.29)	33 417 (25.98)	40 (43.01)	2 (0.11)	1 (0.03)	33 209 (26.68)	33 416 (26.60)	40 (43.01)
30–39	30 478 (24.12)	31 670 (24.63)	28 (30.11)	12 (0.65)	6 (0.20)	30 466 (24.47)	31 664 (25.20)	28 (30.11)
40–49	24 928 (19.73)	25 351 (19.71)	12 (12.90)	19 (1.03)	21 (0.71)	24 909 (20.01)	25 330 (20.16)	12 (12.90)
50–59	18 160 (14.37)	17 559 (13.65)	9 (9.68)	91 (4.93)	75 (2.53)	18 069 (14.51)	17 484 (13.92)	9 (9.68)
60–69	11 170 (8.84)	10 545 (8.20)	2 (2.15)	250 (13.55)	204 (6.88)	10 920 (8.77)	10 341 (8.23)	2 (2.15)
70–79	4 907 (3.88)	4 908 (3.82)	0 (0)	440 (23.85)	476 (16.06)	4 467 (3.59)	4 432 (3.53)	0 (0)
≥ 80	3 483 (2.76)	5 15 (4.01)	2 (2.15)	1031 (55.88)	2181 (73.58)	2452 (1.97)	2 976 (2.37)	2 (2.15)
**Number of Hospital Admissions in Prior Year**								
0	120 063 (95.03)	118,493 (92.14)	93 (100)	1 171 (63.47)	2 105 (71.02)	118 892 (95.50)	116 388 (92.63)	93 (100)
1–2	5 428 (4.30)	9 289 (7.22)	0 (0)	566 (30.68)	735 (24.80)	4 862 (3.91)	8 554 (6.81)	0 (0)
3–5	727 (0.58)	724 (0.56)	0 (0)	101 (5.47)	115 (3.88)	626 (0.50)	609 (0.48)	0 (0)
≥ 6	119 (0.09)	101 (0.08)	0 (0)	7 (0.38)	9 (0.30)	112 (0.09)	92 (0.07)	0 (0)
**Location of COVID-19 Test Collection**								
Hospital	1 228 (0.97)	1 066 (0.83)	0 (0)	41 (2.22)	38 (1.28)	1 187 (0.95)	1 028 (0.82)	0 (0)
ED	8 408 (6.66)	8 423 (6.55)	0 (0)	41 (2.22)	47 (1.58)	8 367 (6.72)	8 376 (6.67)	0 (0)
LTC	1 763 (1.40)	2 879 (2.24)	0 (0)	1 763 (95.56)	2 879 (97.13)	0 (0)	0 (0)	0 (0)
Community	114 938 (90.98)	116,239 (90.38)	93 (100)	0 (0)	0 (0)	114 938 (92.33)	116 239 (92.52)	93 (100)
**Number of Comorbidities**								
0	46,901 (37.12)	39,483 (30.70)	29 (31.18)	76 (4.12)	221 (7.46)	46,825 (37.61)	39,262 (31.25)	29 (31.18)
1+	66,557 (52.68)	83,971 (65.29)	24 (25.81)	1769 (95.88)	2740 (92.44)	64,788 (52.04)	81,231 (64.65)	24 (25.81)
No contacts*	12,879 (10.19)	5153 (4.01)	40 (43.01)	0 (0.00)	3 (0.10)	12,879 (10.35)	5150 (4.10)	40 (43.01)
**Comorbidities (Top 5)**								
Depression	24,959 (16.56)	42,766 (22.58)	9 (25.71)	900 (10.94)	1414 (11.78)	24,059 (16.89)	41,352 (23.31)	9 (25.71)
Hypertension, uncomplicated	23,042 (15.29)	20,798 (10.98)	4 (11.43)	947 (11.51)	1511 (12.59)	22,095 (15.51)	19,287 (10.87)	4 (11.43)
Rheumatoid Arthritis	17,510 (11.62)	22,754 (12.01)	7 (20.00)	484 (5.88)	820 (6.83)	17,026 (11.95)	21,934 (12.37)	7 (20.00)
Diabetes	13,370 (8.87)	11,967 (6.32)	3 (8.57)	695 (8.45)	821 (6.84)	12,675 (8.90)	11,146 (6.28)	3 (8.57)
Chronic Pulmonary Disorders	10,083 (6.69)	12,517 (6.61)	3 (8.57)	415 (5.04)	619 (5.16)	9668 (6.79)	11,898 (6.71)	3 (8.57)

*No contacts indicates no records available to assess comorbidities

### Primary outcomes

#### Acute Hospitalizations and ambulatory care visits

Overall acute care hospitalization was 5.9% (n = 15,088) of which 52.6% were among males and 4.3% were among LTC residents (Table 2). Almost 46.0% of hospitalizations occurred among individuals older than 60 years of age. In the Poisson model exploring variables associated with hospitalization in LTC residents, male sex compared to female, was more likely to be associated with hospitalization (RR 1.51, 95%CI 1.31–1.75, p < 0.001), as was ≥ 6 hospitalizations in the prior year (RR 1.90, 95%CI 1.05–3.42, p = 0.03), comorbidities of complicated hypertension (RR 2.39, 95%CI 1.40–4.08 p < 0.001) and comorbidity of diabetes (RR 1.52, 95%CI 1.29–1.81, p < 0.001).

**Table 2:** Outcomes among all incident cases of COVID-19 between March 1, 2020 and December 15, 2021, stratified by sex and LTC residency

	All Individuals (N = 255 037)			Individuals in LTC (N = 4 809)		Individuals not in LTC (N = 250 288)			P-value*
Acute Care Hospitalization Within 60 days									
Age Group (years)	Male (N = 126 337)	Female (N = 128 607)	Not Documented (N = 93)	Male (N = 1 845)	Female (N = 2 964)	Male (N = 124 492)	Female (N = 125 643)	Not Documented (N = 93)	
18–59	3 865 (3.06)	4 012 (3.12)	1 (1.08)	18 (0.98)	23 (0.78)	3 847 (3.09)	3 989 (3.17)	1 (1.08)	< 0.001
60–79	2 865 (2.27)	2 060 (1.60)	0 (0)	152 (8.24)	101 (3.41)	2 713 (2.18)	1 959 (1.56)	0 (0)	0.003
≥ 80	1 205 (0.95)	1 080 (0.84)	0 (0)	163 (8.83)	188 (6.34)	1 042 (0.84)	892 (0.71)	0 (0)	< 0.001
**Unplanned Ambulatory Visit Within 60 Days**									
**Age Group (years)**									
18–59	15 331 (12.14)	18 108 (14.08)	5 (5.38)	21 (1.14)	31 (1.05)	15 310 (12.30)	18 077 (14.39)	5 (5.38)	0.003
60–79	4 986 (3.95)	4 299 (3.34)	0 (0)	181 (9.81)	140 (4.72)	4 805 (3.86)	4 159 (3.31)	0 (0)	< 0.001
≥ 80	1 560 (1.23)	1 549 (1.20)	0 (0)	216 (11.71)	293 (9.89)	1 344 (1.08)	1 256 (1)	0 (0)	< 0.001
**Patient Status At 60 Days**									
Death	1 973 (1.56)	1 534 (1.19)	0 (0)	549 (29.76)	636 (21.46)	1 424 (1.14)	898 (0.71)	0 (0)	< 0.001
In ICU/CV-ICU	47 (0.04)	21 (0.02)	0 (0)	0 (0)	0 (0)	47 (0.04)	21 (0.02)	0 (0)	0.485
In Hospital	458 (0.36)	396 (0.31)	0 (0)	27 (1.46)	35 (1.18)	431 (0.35)	361 (0.29)	0 (0)	< 0.001
In LTC	175 (0.14)	181 (0.14)	0 (0)	123 (6.67)	139 (4.69)	52 (0.04)	42 (0.03)	0 (0)	< 0.001
Discharged	6 013 (4.76)	5779 (4.49)	1 (1.08)	12 (0.65)	2 (0.07)	6 001 (4.82)	5 777 (4.60)	1 (1.08)	< 0.001
Community	117 671 (93.14)	120 696 (93.85)	92 (98.92)	1 134 (61.46)	2 152 (72.60)	116 537 (93.61)	118 544 (94.35)	92 (98.92)	< 0.001
**All-cause Mortality within 60 days**									
**Age Group (years)**									
18–59	243 (0.19)	132 (0.10)	0 (0)	11 (0.60)	6 (0.20)	232 (0.19)	126 (0.10)	0 (0)	< 0.001
60–79	787 (0.62)	494 (0.38)	0 (0)	141 (7.64)	117 (3.95)	646 (0.52)	377 (0.30)	0 (0)	< 0.001
≥ 80	943 (0.75)	908 (0.71)	0 (0)	397 (21.52)	513 (17.31)	546 (0.44)	395 (0.31)	0 (0)	< 0.001

*P-value calculated comparing LTC to non LTC residents overall using Chi-squared analyses

**Fig. 1:**
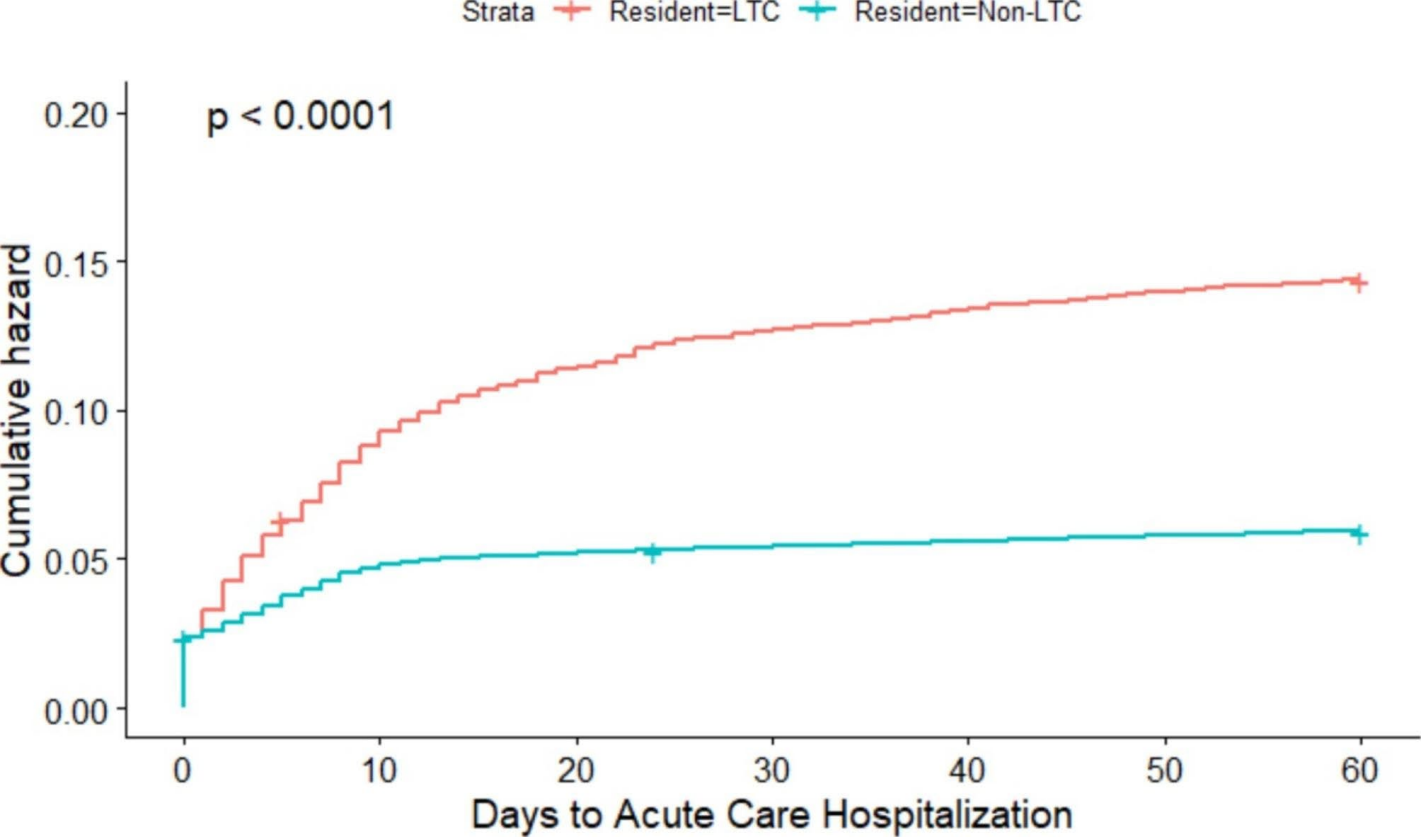
Risk of acute care hospitalization in days since COVID diagnosis for LTC residents versus non-LTC residents

The overall mean time to acute care hospitalization was 7.8 days (standard deviation, SD 13.0) and median time was 3 days (IQR 8). The median time to acute care hospitalization was two days earlier for males (2 days) compared to females (4 days) but was not significantly different. The median time for LTC residents was 6 days (IQR 13) compared to 3 days (IQR 8) for non-LTC residents (p < 0.0001) (Fig. 1). Overall, 18.0% of COVID-19 confirmed cases had an ambulatory care visit within 60 days with the majority occurring in the first 10 days.

#### All-cause Mortality

**Fig. 2:**
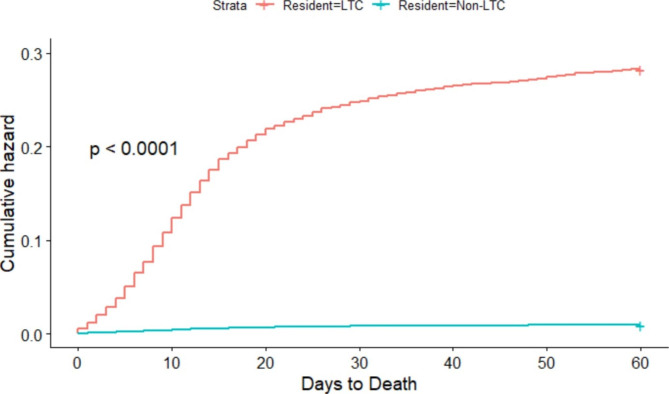
Risk of death in days since COVID diagnosis for LTC residents versus non-LTC residents

Between March 2020 and December 2021 there were 3,507 deaths within 60 days, for an overall all-cause mortality of 1.4%, with 56.3% occurring among males (Table 2). Of those who died, 36.5% occurred among individuals aged 60–79 years, and 52.8% among individuals 80 + years. The median time to death (excluding one record with an incorrect death date) was 12 days (IQR 14) and the mean time was 15.2 days (SD 12.8). The median time to death was one day earlier for males (11 days) compared to females (12 days). The median time to death for LTC residents (11 days, IQR 12) was one day earlier than non-LTC residents, (12 days, IQR 15) (p < 0.0001) (Fig. 2). At 60 days, 98.1% were alive and not in LTC, hospital, or the ICU. However, at 60 days, 24.6% of the 4,809 LTC residents with COVID-19 had died. Among hospitalized patients, 2,017 (13.4%) died within 60 days, with deaths occurring mostly among males (61.6%) and within hospital (94.4%). When using Chi-squared analyses to compare mortality rates amongst LTC and non-LTC residents there was significantly higher mortality in those in LTC (0.25% vs. 0.01%, p < 0.001). When stratified by age, this relationship remained true in the youngest age group spanning 18–59 years (0.07% vs. 0.002%, p < 0.001), the age group of 60–79 years (0.19% vs. 0.03%, p < 0.001) and the oldest age group of ≥ 80 years (0.28% vs. 0.17%, p < 0.001). Mortality rates increased with advancing age in both LTC and non-LTC residents.

The Poisson model demonstrated that in LTC residents, male sex had higher mortality (RR 1.52, 95%CI 1.38–1.68, p < 0.001), as did comorbidities of diabetes (RR 1.18, 95%CI 1.03–1.34, p = 0.01), chronic pulmonary disease (RR 1.21, 95%CI 1.02–1.43, p = 0.02), and obesity (RR 1.64, 95%CI 1.11–2.43, p = 0.01).

### Secondary outcomes

#### In-hospital healthcare utilization and in-hospital mortality

ICU admission was required for 2,946 (19.5%) of all hospitalized cases, while cardiovascular (CV)-ICU admission was only required for 143 individuals (0.9%) (Table 3). Males were more commonly admitted to the ICU (63.8% vs. 36.2%) or CV-ICU (75.5% vs. 24.5%) compared to females. The overall median time to the first ICU admission was 4 days (IQR 7), whereas the median time to first CV-ICU visit was 12 days (IQR 25). The median time to the first ICU visit for males was the same as for females (both were 4 days). Of the 645 LTC residents that required hospitalization, 30 (4.7%) required an ICU admission compared to 2,916 (20.2%) of 14,443 non-LTC residents. The median time to the first ICU visit for LTC residents was 3.5 days later than for non-LTC residents (7.5 days vs. 4 days). No LTC residents required a CV-ICU admission. Nineteen (2.9%) hospitalized LTC residents received mechanical ventilation compared to 2,137 (14.8%) of non-LTC residents. Overall median time to the first ventilation was 4 days (IQR 8), with LTC residents having a longer median time to ventilation compared to non-LTC residents (7 vs. 4 days). The median LOS of the first hospitalization within 60 days for non-LTC residents was 5 days (IQR 8), and 8 days (IQR 12) for LTC residents. In-hospital mortality increased with advanced age particularly amongst those in LTC (Table 3).

**Table 3:** Hospitalization outcomes among incident cases of COVID-19 between March 1 2020 and December 15 2021 stratified by sex and LTC residency

	All Individuals (N = 15 088)		Individuals in LTC (N = 645)		Individuals not in LTC (N = 14 443)		P-value*
Acute Care Hospitalization Within 60 days							
Age Group (years)	Male (N = 7935)	Female (N = 7152)	Male (N = 333)	Female (N = 312)	Male (N = 7602)	Female (N = 6840)	
18–59	3865 (48.71)	4012 (56.10)	18 (5.41)	23 (7.37)	3847 (50.61)	3989 (58.32)	
60–79	2865 (36.11)	2060 (28.80)	152 (45.65)	101 (32.37)	2713 (35.69)	1959 (28.64)	
≥ 80	1205 (15.19)	1080 (15.10)	163 (48.95)	188 (60.26)	1042 (13.71)	892 (13.04)	
**ICU Admission Within 60 Days**							
**Age Group (years)**							
18–59	944 (11.90)	598 (8.36)	5 (1.50)	4 (1.28)	939 (12.35)	594 (8.68)	0.851
60–79	846 (10.66)	435 (6.08)	10 (3.00)	5 (1.60)	836 (11.00)	430 (6.29)	< 0.001
≥ 80	91 (1.15)	32 (0.45)	4 (1.20)	2 (0.64)	87 (1.14)	30 (0.44)	0.001
**CV-ICU Admission Within 60 Days**							
**Age Group (years)**							
18–59	56 (0.71)	14 (0.20)	0 (0)	0 (0)	56 (0.74)	14 (0.20)	1.000
60–79	42 (0.53)	20 (0.28)	0 (0)	0 (0)	42 (0.55)	20 (0.29)	0.120
≥ 80	10 (0.13)	1 (0.01)	0 (0)	0 (0)	10 (0.13)	1 (0.01)	0.319
**Mechanical Ventilation Within 60 days**							
**Age Group (years)**							
18–59	673 (8.48)	452 (6.32)	5 (1.50)	4 (1.28)	668 (8.79)	448 (6.55)	0.236
60–79	632 (7.96)	316 (4.42)	6 (1.80)	4 (1.28)	626 (8.23)	312 (4.56)	< 0.001
≥ 80	56 (0.71)	27 (0.38)	0 (0)	0 (0)	56 (0.74)	27 (0.39)	< 0.001
**Median (IQR) Length of Stay for First Hospitalization**							
**Age Range**							
18–59	4.00 (7.00)	3.00 (5.00)	7.50 (8.50)	10.00 (23.50)	4.00 (7.00)	3.00 (5.00)	< 0.001
60–79	7.00 (11.00)	6.00 (9.00)	9.00 (13.00)	10.00 (15.00)	6.00 (11.00)	6.00 (9.00)	< 0.001
≥ 80	8.00 (12.00)	8.00 (12.00)	7.00 (9.00)	7.00 (11.00)	8.00 (12.00)	8.00 (12.00)	0.027
**In-Hospital Mortality Within 60 Days**							
**Age Range**							
18–59	157 (1.98)	92 (1.29)	6 (1.80)	4 (1.28)	151 (1.99)	88 (1.29)	< 0.001
60–79	542 (6.83)	302 (4.22)	58 (17.42)	34 (10.90)	484 (6.37)	268 (3.92)	< 0.001
≥ 80	480 (6.05)	332 (4.64)	91 (27.33)	81 (25.96)	389 (5.12)	251 (3.67)	< 0.001
**Patient Status At 60 Days**							
Death	1242 (15.65)	775 (10.84)	171 (51.35)	136 (43.59)	1071 (14.09)	639 (9.34)	< 0.001
In ICU/CV-ICU	47 (0.59)	21 (0.29)	0 (0)	0 (0)	47 (0.62)	21 (0.31)	0.148
In Hospital	458 (5.77)	396 (5.54)	27 (8.11)	35 (11.22)	431 (5.67)	361 (5.28)	< 0.001
In LTC	175 (2.21)	181 (2.53)	123 (36.94)	139 (44.55)	52 (0.68)	42 (0.61)	< 0.001
Discharged	6013 (75.78)	5779 (80.80)	12 (3.60)	2 (0.64)	6001 (78.94)	5777 (84.46)	< 0.001

*P-value calculated comparing LTC to non LTC residents overall using Chi-squared analyses for rates and Wilcoxin tests for medians

In the Poisson model for ICU admission in LTC residents decreased ICU admission was associated with increasing age of ≥ 80 years (RR 0.04, 95%CI 0.00-0.34, p < 0.001). Increased ICU admission in LTC residents was associated with ≥ 1 comorbidity (RR 4.80, 95%CI 1.07–21.45, p-0.04), and comorbidity of complicated hypertension (RR 8.45, 95%CI 2.63-15., p < 0.001). An increase in mechanical ventilation in LTC residents was associated with > 6 more hospital visits in the prior year (RR 10.97, 95%CI 2.99–40.31, p < 0.001).

## Discussion

This descriptive population-based study is an overview of healthcare utilization and outcomes among incident cases of COVID-19 in Alberta, Canada between March 1, 2020 and December 15, 2021, stratified by age, sex and LTC residency. Our findings are similar to a previous study from Alberta and Ontario that demonstrated over the time period of March 1, 2020 to June 30, 2021 the majority of cases were in adults < 65 years of age [15]. This same study also demonstrated mortality of 1.5% within 30 days of diagnosis of COVID-19 which is very similar to our finding of 1.4% within 60 days of diagnosis and a LOS of 6 days for those patients in Alberta, similar to our finding of 5 days and 8 days for non LTC and LTC residents, respectively [15]. This suggests that the majority of deaths are likely happening within 30 days of diagnosis of COVID-19. Hospital admissions within 30 days of diagnosis were 5.5% compared to our finding of 5.9% within 60 days of diagnosis, again implying the biggest impact on inpatient healthcare utilization occurs shortly after diagnosis of COVID-19 [15]. Our finding of time to hospitalization of 7.8 days from diagnosis is in keeping with prior work summarizing the epidemiology of COVID-19 which suggest that if patients require hospitalization it typically occurs within a week of diagnosis and symptoms [12].

Our findings are consistent with prior work that demonstrated negative outcomes of COVID-19 on the LTC population [8, 9]. We found a 60-day mortality of 24.6% for those who were LTC residents, as opposed to 1.4% mortality in our overall population, and 13.4% mortality for those who were admitted to hospital. While a smaller percent of LTC residents went to ICU (4.7%) as compared to non LTC residents (20.2%) and patients ≥ 80 years of age were less likely to go to ICU, this may reflect different goals of care in these two patient groups as LTC residents are less likely to request and qualify for ICU care, particularly at advanced age. We explored the relationship between sex, age, number of prior hospitalizations and comorbidities for poor outcomes in the LTC residents, and did find that the most common comorbidities amongst LTC residents included chronic pulmonary disorders which were linked to poor outcomes in LTC residents in Ontario in a recent study [9]. In our study chronic pulmonary disorders was associated with higher relative risk of mortality.

Depression was a common comorbidity in both those who were and were not residents of LTC. Prior work has demonstrated that those with mood disorders are more at risk of hospitalization and death due to COVID-19 [16]. There are multiple reasons why this relationship between COVID-19 and mood disorders may exist including immune dysregulation in those with mood disorders (from the disorder itself or medications used to treat it) and social determinants of health (e.g. those with mood disorders may have more difficulty accessing appropriate care or taking health precautions) [16]. While our study does describe an association between depression and positive COVID-19 test results, when we explored depression as a variable in our adjusted Poisson model for poor outcomes in LTC residents, it was not significant. There is growing evidence that depression, in addition to being linked to a diagnosis of COVID-19 may be associated with long COVID-19 [17]. While our study was not able to explore this, this should be further investigated as this has substantial implications on healthcare utilization [18].

We found that of those that died, 56.3% were male. Once hospitalized for COVID-19 a greater proportion of males made up the group that died (61.6%). Additionally, men were more likely to go to ICU than females (63.8% vs. 36.2%) and CV-ICU (75.5% vs. 24.5%). Prior work has demonstrated that men are more likely to die from COVID-19 compared to women, even when factoring in other medical comorbidities and age, and have more severe outcomes including ICU admissions [19, 20]. This higher risk of severe disease and death is in keeping with the finding that respiratory tract infections are more severe in men and can lead to higher mortality amongst men, suggesting a potential underlying biological mechanism [19, 20]. Gender-related social factors such as smoking, healthcare seeking behaviours and some comorbidities may impact outcomes of COVID-19 and provide some explanation for the morbidity and mortality in males, however, as similarities have emerged across different geographic locations and cultures it again points to biological risk determinants [19].

Finally, while there has been prior work done on outpatient care for patients in the era of COVID-19, [21, 22] there is relatively little information on unexpected ambulatory care visits for patients with COVID-19. Our study demonstrated that in the younger age group of 18–59 years with positive COVID-19 tests, 12.1% of males and 14.1% females had an unplanned ambulatory care visit within 60 days of diagnosis. While the focus on impacts of COVID-19 is often on acute care hospitalizations the majority of patients with a positive diagnosis are under the age of 60 and the burden on the healthcare system including ambulatory care should also be considered and explored.

These findings are important for pandemic preparedness and planning health policy solutions for both COVID-19 and future emerging viruses. For example, our findings suggest that the first 30 days following COVID-19 diagnosis are when the majority of hospitalizations occur, and the first 10 days are where the majority of unplanned ambulatory visits to urgent care or emergency departments arise, suggesting a need for close follow up during the acute period.

Our study has several strengths, it is population-based and allowed us to explore all patients with a positive COVID-19 test in Alberta for the majority of the COVID-19 pandemic timeline. We also did a 60-day follow up to provide a longer timeline for a more robust assessment of outcomes following a COVID-19 diagnosis. We also were able to stratify our population by sex which allowed us to confirm prior results that demonstrated worse outcomes for males compared to females. We were able to explore the LTC population who have been substantially impacted by COVID-19. Finally, we were able to determine the proportion of patients who had an unplanned ambulatory care visit within 60 days of diagnosis of COVID-19.

There are also several limitations. Administrative data relies on coding which can often result in human errors. However, given the prohibitive resource expenditure associated with primary data collection in an entire population the use of administrative data did allow for the success of this work. Vaccines were introduced during the study period, which may have influenced the severity of outcomes over time, but vaccine data was not available for the current study. However, this will be explored in future studies by our group. Testing policies for COVID-19 have changed during the study period in Alberta several times, which may change severity of cases that were identified and influenced the identified proportion of those hospitalized and those who required unplanned ambulatory visits. A detailed analysis exploring the impacts of changing testing strategies should be considered in the future and overlaid with these data to assess its impact on hospitalization and ambulatory visits. The influence of changing testing strategies on patients that were hospitalized is somewhat mitigated as patients admitted to hospital were generally tested for COVID-19 even if not having typical symptoms. Finally, the only variables available for our models were sex, age, comorbidities and number of prior hospitalizations. Other variables that may have been relevant to outcomes such as socioeconomic status and geographic location were not able to be explored.

## Conclusions

Our work was able to stratify all patients in Alberta who tested positive for COVID-19 in LTC as well as those who had an unplanned ambulatory visit within 60 days of diagnosis of COVID-19. Future work should explore the healthcare utilization of patients who tested positive for COVID-19 including costs associated with inpatient hospitalizations and ambulatory care visits. Younger patients (< 60) were more likely to seek ambulatory care and made up the majority of those who were diagnosed with COVID-19. Therefore, while they may not require hospitalization there is still a large burden on the healthcare system which is necessary to quantify. Additionally, further work should explore the impact of vaccines on the outcomes of these patients and the timeline should be further stratified into the various COVID-19 waves to delineate any differences.

## Data Availability

The data that support the findings are available from Alberta Health Services but restrictions apply to the availability of these data which were used under license for the current study, and so are not publicly available. Data are however available from the authors upon reasonable request and with permission of Alberta Health Services.

## List of Abbreviations

COVID-19: Coronavirus Disease of 2019
LTC: Long-term care
NAAT: Nucleic acid amplification test
ACARD: Alberta COVID-19 Analytics and Research Database
PHN: Personal healthcare number
APL: Alberta Precision Laboratories
AHS: Alberta Health Services
EDW: Enterprise Data Warehouse
DAD: Discharge Abstract Database
ACCIS: Alberta Continuing Care Information System
ICD-10-CA: International Statistical Classification of Diseases and Related Health Problems, Tenth Revision, Canada
ICU: Intensive care unit
CVICU: Cardiovascular intensive care unit
LOS: Length of stay
SD: Standard deviation
IQR: Interquartile range
